# Utilization of Purple Napier Grass Silage on Milk Quality and Blood Antioxidant Activity in Lactating Dairy Goats

**DOI:** 10.3390/ani14223209

**Published:** 2024-11-08

**Authors:** Weerada Meethip, Siwaporn Paengkoum, Narawich Onjai-uea, Sorasak Thongpea, Nittaya Taethaisong, Jariya Surakhunthod, Pramote Paengkoum

**Affiliations:** 1 School of Animal Technology and Innovation, Institute of Agricultural Technology, Suranaree University of Technology, Muang, Nakhon Ratchasima 30000, Thailand; weerada.m508@gmail.com (W.M.); sorasak.t@sut.ac.th (S.T.); iszy.nittaya@gmail.com (N.T.); lt1jariya.su@gmail.com (J.S.); 2 Program in Agriculture, Faculty of Science and Technology, Nakhon Ratchasima Rajabhat University, Muang, Nakhon Ratchasima 30000, Thailand; siwaporn.p@nrru.ac.th; 3 Department of Animal Science, Faculty of Agriculture at Kamphaeng Saen, Kasetsart University, Kamphaeng Saen Campus, Nakhon Pathom 73140, Thailand; ulbare@gmail.com

**Keywords:** sweet grass, Napier Pakchong-1, purple Napier, silage, rumen fermentation, milk quality, blood antioxidant activity, lactating goats

## Abstract

In this study, our subjects were 18 female crossbred Saanen lactating goats; the goats weighed approximately 43.25 ± 2.35 kg and had been producing milk for 14 ± 2 days. They were assigned to three equally sized experimental groups based on the cycle of the lactation period. In accordance with the randomized complete block design (RCBD), six animals were randomly assigned to the treatments in each block. There were three treatments: (1) control = Napier Pakchong-1 grass silage (NPS); (2) = sweet grass silage (SGS); and (3) = purple Napier silage (PNS). Our findings show that the goats fed with purple Napier grass silage had a higher DM intake (1805.97 g/day) than those that underwent a different treatment (1312.76 and 1443.43 g/day). The goats fed with purple Napier grass silage had higher protein digestibility (79.85) than those fed with Napier Pakchong-1 and sweet grass silage. There were significant differences in the ruminal ammonia nitrogen and blood urea nitrogen. Nevertheless, purple Napier grass silage significantly increased butyric acid 2 and 4 h after feeding and increased the number of microorganisms. In the dairy goats fed purple Napier grass silage, we observed a reduction in protozoa and methanogen populations. The levels of antioxidants shown by the plasma indices differed in the goats fed with purple Napier grass silage and showed enhanced milk composition, higher levels of total antioxidants, superoxide dismutase and glutathione peroxidase in the plasma, reduced protozoa methanogen, and a decreased level of malondialdehyde in the plasma. This study highlights that purple Napier grass silage has the potential to be a beneficial source of roughage for lactating dairy goats.

## Simple Summary

Purple Napier grass silage is a feed widely used in the tropics and subtropics for dairy and meat production that is useful to ruminants. Purple Napier grass silage, recognized for its antioxidant properties, plays a significant role in producing various compounds, including polyphenols. The increased use of purple Napier grass silage has led to significant differences in lactose levels, somatic cell count, glutathione peroxidase, total antioxidant capacity, superoxide dismutase, and performance. Consuming feed enriched with these bioactive compounds naturally and effectively maintains a healthy antioxidant balance. In addition to improving animal performance, this approach also ensures the safety of certain animal products that are intended for human consumption.

## 1. Introduction

Thailand, with its tropical climate, has an extended period of unusually dry weather from November to March, which distinguishes it from other regions [1]. As silage production has become more prevalent, it is now an essential component of ruminant feed [2]. However, it has been found that there are many types of animal feed plants that can grow well under dry conditions [3], and these plants can be used as an alternative feed for ruminant production. This is considered one of the methods that can help reduce the cost of raising animals, particularly the cost of feed [4]. As a result, farmers have increased their income from raising animals by using local plants or raw materials for maximum benefit [5]. Furthermore, these plants can be preserved for use in times of shortage or during the dry season. Silage made of these grasses preserves them for use in times of shortage and is a source of good-quality roughage that farmers can grow and make themselves, thereby helping to reduce costs [6]. Recent in vivo studies have reported that the microbial population in the rumen fluid of goats fed with purple Napier grass [3] containing anthocyanin enhanced the acetate-to-propionate ratio.

Napier Pakchong-1 (*Pennisetum purpureum* × *Pennisetum americanum* cv. Pakchong 1) is a tall pasture variety and a high-yielding grass. Therefore, Napier grass accounts for up to 80% of the forage consumed by cows in many tropical and subtropical countries [7]. This type of grass contains plant pigment anthocyanins, which have a variety of uses, such as providing color to food and reducing the risk of disease. In the tropics, goats are fed a diet rich in anthocyanin, which can boost the antioxidant capacity of their rumen. Moreover, Onjai-uea et al. [3] reported that purple Napier grass has the highest anthocyanin content. Sweet grass is a small Napier grass with exceptional nutritional values, is highly palatable and adaptable, and is easy to manage and harvest [8]. SG (sweet grass) is a high-quality forage for improving rumen fermentation and decreasing methane production, which are factors that are particularly important under the conditions of global climate change [9].

Onjai-uea et al. [10] found that feeding 100% purple Napier grass silage could improve various parameters, including milk composition. The researchers found minimal malondialdehyde in plasma and milk [11].

Moreover, there is a lack of research on feeding sweet grass, Napier Pakchong-1 grass and purple Napier grass in silage to ruminant animals. The aim of this study was to investigate the effects of the utilization of purple Napier grass silage on milk quality and blood antioxidant activity in lactating dairy goats.

## 2. Materials and Methods

### 2.1. Designing an Animal Experiment and Diets

The experiment used eighteen female crossbred Saanen lactating goats, with an average weight of 43.25 ± 2.35 kg. These goats were divided into three groups based on their lactation period. Using a randomized complete block design (RCBD), six animals were randomly assigned to one of the treatments within each block. The trial lasted for 90 days; after 14 days of adaptation to feeding, three treatments were conducted, as follows: (1) control, = Napier Pakchong-1 grass silage; (2) = sweet grass silage; (3) = purple Napier grass silage. The dairy goats received a mixed total feed ration (TMR) of roughage with a concentrate ratio of 60:40 (Table 1 and Table 2).

### 2.2. Animal Management

The goats were kept in clean, dry pens. The feeding was at 07.00 and 16.00, with diets referred to as total mixed rations (TMRs). The goats were weighed at the beginning and end of the experiment period in order to measure their growth performance.

### 2.3. Chemical Analysis of Feeds and Milk

The finely ground sample was then analyzed for its total chemical composition. The total nitrogen (N) was determined using the Kjeldahl method, and the crude protein (CP) was calculated by multiplying the N content by 6.25, in accordance with previous studies. The quantification of the ether extract (EE) and the ash contents followed the guidelines established by the authors of AOAC [12]. The estimation of the neutral detergent fiber and acid detergent fiber followed the methods outlined by Van Soest et al. [13].

The milk samples were divided into two portions. One portion was preserved with bronopol tablets (D&F Control System Inc., San Ramon, CA, USA) at 4 °C until the analysis of milk composition, and the calculation of fat-corrected milk (3.5% FCM) for the milk production was performed using the formula 3.5% FCM = kilograms of milk yield × [0.432 + 0.162 × (fat%)].

### 2.4. Procedures for Feed

Routine weighing of the provided and refused feed was carried out before the morning feeding. The amount of milk yield from the dairy goats was recorded once a day, and individual goat milk samples, representing the morning milking quantity from each goat, were taken.

### 2.5. Fecal Sampling

Fecal samples were collected from each individual goat using a collection procedure involving dry matter, ether extract, and crude protein. The procedure followed the protocol outlined by of Wrolstad et al. [14]. ADF and NDF were determined in accordance with the process detailed in the study by Van Soest [15].

### 2.6. Urine Sampling Procedures

Total urine collection was obtained from each animal. The samples were obtained directly using collector funnels coupled to hoses, which directed the urine to a plastic container for each recipient; 100 mL of 10% H_2_SO_4_ was used to acidify (final pH < 3) the collected urine. The urine volume was quantified at the end of each 24 h period, and subsamples were filtered through cheesecloth layers and immediately diluted with 40 mL of 0.036 N H_2_SO_4_ solution. The urine samples were composited per period for each animal in quantities that were proportional to those of the 3 sampling days. Composite samples were stored at −20 °C for further analyses using the procedure detailed by Dos Santos et al. [16].

### 2.7. Apparent Digestibility

The acid insoluble ash (AIA) approach was used to calculate the apparent nutrient digestibility (%). The calculation, as provided by Furuichi et al. [17], is as follows: apparent nutritional digestibility (%) = 100 − ((100 × %AIA in diet × %AIA in fecal)/(%AIA in fecal × %AIA in diet)).

### 2.8. Blood Sampling Procedures

Eighteen goats’ blood samples were taken over the trial’s last two days. Furthermore, Vacuette^®^ tubes (Greiner Bio-One, Greiner Bio-One GmbH, Bad Haller Str., Kremsmünster, Austria) containing K3-EDTA were used to draw blood samples (about 3 mL each) from the jugular vein at 0, 2, and 4 h after eating.

### 2.9. Assessment of Antioxidant Activity in Plasma

At 0, 2, and 4 h after feeding, blood samples were taken from the jugular vein and placed into a single 10 mL vacuum tube containing heparin. Once the blood sample was centrifuged at 3500× *g* for 20 min at 4 °C using an Allegra R X-30R centrifuge (Beckman Coulter, Life Sciences Division Headquarters, Indianapolis, IN, USA), it was transferred to a 1.5 mL tube and kept at 20 °C until the antioxidant activity enzymes in the plasma were examined.

### 2.10. Rumen Fermentation Measurements

Ruminal fluid was collected at 0, 2, and 4 h after feeding using a stomach tube attached to a vacuum pump at the end of the trial. The samples of rumen fluid were anaerobically filtered through four layers of cheesecloth, and the pH values of the samples were promptly determined using a portable pH meter. The samples of ruminal fluid were then split into two halves. The first part of the ruminal fluid samples was obtained following the protocol described below. The second part was utilized to detect the ammonia nitrogen measurement, following the procedure outlined by Wrolstad et al. [14]. After that, the strained rumen fluid was transferred right away to the lab and put in a sterile thermos flask. The quantities of VFAs in the filtrates were measured by gas chromatography (Agilent 6890 GC, Agilent Technologies, Santa Clara, CA, USA) using a silica capillary column (30, 250, 0.25 m).

### 2.11. Extraction of DNA and Real-Time PCR Analysis

To extract the ruminal fluid sample for community DNA analysis, a commercial kit (QIAGEN, Inc., Hilden, Germany) was used. The rumen microbial populations studied in this experiment included total bacteria, methanogens, and protozoa. Butyrivibrio fibrisolvens, Fibrobacter succinogenes, Ruminococcus flavefaciens, Streptococcus bovis, and Ruminococcus albus were analyzed using the real-time PCR technique. For the assays of the PCR cycle, the conditions were as follows: an initial denaturation at 94 °C for 2 min, followed by 55 cycles at 94 °C for 30 s, 63 °C for 40 s, and 72 °C for 35 s for primer annealing and product elongation.

### 2.12. Statistical Analysis

The effect of the diet on various factors, including feed intake, milk yield, milk composition, and antioxidant activity in the plasma of lactating dairy goats, was examined using repeated measures and modeled with the PROC MIXED procedure of Statistical Analysis System software, version 9.4, using a randomized complete block design (RCBD). To ascertain significant differences (*p* < 0.05) among the treatments, the Tukey–Kramer test was used, following the approach described by Steel et al. [18].

## 3. Results

### 3.1. Feed Intake and Nutrient Intake

The effect of the utilization of purple Napier grass silage on growth performance, feed intake and nutrient intake in dairy goats is shown in Table 3. The goats fed with purple Napier grass silage had the highest feed intake (g DM/d, % BW, and g/kg BW^0.75^) and nutrient intake (*p* < 0.05), final weight, weight change, and milk production.

### 3.2. Nutrient Digestibility

The effect of the utilization of purple Napier grass silage on nutrient digestibility in dairy goats is shown in Table 4. The goats fed with purple Napier grass silage had the highest levels of DM, OM, CP, NDF, and ADF, which was significantly higher (*p* < 0.05).

### 3.3. Nitrogen Utilization

The effect of the utilization of purple Napier grass silage on nitrogen utilization in dairy goats is shown in Table 5. The goats fed with purple Napier grass silage had the highest nitrogen utilization (*p* < 0.05) compared to that of the NPS and SGS treatments.

### 3.4. Rumen Fermentation

The effect of the utilization of purple Napier grass silage on rumen fermentation is shown in Table 6. The pH parameters did not show a significant difference (*p* > 0.05), and the NH_3_-N, and BUN values were not different between the groups at 0 h (*p* > 0.05). There were significant differences (*p* < 0.05) in the NH_3_-N and BUN values among the treatments at 2 and 4 h after feeding. The goats fed NPS had increased NH_3_-N and BUN values compared to the other treatments.

### 3.5. Volatile Fatty Acid

The effect of the utilization of purple Napier grass silage on volatile fatty acids in the dairy goats is shown in Table 7. There were no differences in the volatile fatty acids between the groups (*p* > 0.05) at 0 h, while there were significant (*p* < 0.05) variations in the volatile fatty acids among the treatments at 2 and 4 h after feeding. However, the goats fed with PNS had significantly higher C_2_, C_3_, C_4_, (C_2_/C_3_), and TVFA than in the other treatments.

### 3.6. Microbial Population

The effect of the utilization of purple Napier grass silage on the microbial population in the dairy goats is shown in Table 8. There were significant variations in the microbial communities at 2 and 4 h after feeding (*p* < 0.01). The dairy goats fed with PNS showed higher levels of total bacteria and had reduced protozoa and methanogen at 2 and 4 h after feeding, respectively, compared to the other treatments.

### 3.7. Milk Quality

The effect of the utilization of purple Napier grass silage on milk yield and composition is shown in Table 9. There were significant differences (*p* < 0.01). The goats fed with purple Napier grass silage had the highest production in terms of milk yield (kg/day), 3.5% FCM (kg/day), protein (g/day), fat, lactose (g/day), milk solids not fat (g/day), total solids (g/day), and milk composition in terms of protein (%), fat (%), lactose (%), milk solids not fat (%), and total solids (%) (*p* < 0.01). The goats fed with purple Napier grass silage had a reduced cell count of somatic cells in their milk (SCC) (*p* < 0.01).

### 3.8. Plasma Antioxidant

The effect of the utilization of purple Napier grass silage on plasma antioxidants in dairy goats is shown in Table 10. There were no significant differences at 0 h (*p* > 0.05). The total antioxidants, SOD, and GSH increased at 2 and 4 h after the goats were fed with purple Napier grass (*p* < 0.01). The goats fed with purple Napier grass silage had low levels of malondialdehyde (MDA) at 2 and 4 h (post-feeding) (*p* < 0.01).

## 4. Discussion

### 4.1. Feed Intake and Nutrient Intake

The goats fed with purple Napier grass silage had high feed intake, % BW, g/kg BW^0.75^, final weight, weight change, and milk production, demonstrating that providing purple Napier grass silage can promote palatability for goats; this is because of the anthocyanin content in purple Napier grass silage. Onjai-Uea et al. [3] found that meat goats fed with purple Napier grass had the highest final weight, the highest growth rate, the greatest consumption of roughage, and the highest level of dry matter intake. The study by Bureenok et al. [19] determined that fermented Napier grass had a high feed intake when supplemented with molasses. In [20,21], the authors reported that purple neem leaf supplementation at a rate of 6% improved the palatability and resulted in a high feed intake. The study by Suong et al. [22] found that anthocyanins from fermented corn had no effect on feed edibility among animals. Furthermore, in the current research, there were no negative effects caused by the use of Napier Pakchong-1 grass silage and sweet grass silage. Both of these grasses are a good source of fermented roughage for ruminants and show a good level of feed intake in dairy goats. In general, many factors influence ruminant feed intake, including the feed’s physical characteristics, the raw materials used for mixing, and the nutritional value of various feeds.

### 4.2. Nutrient Digestibility

The effect of sweet grass, Napier Pakchong-1 and purple Napier grass silage on nutrient digestibility was shown in the dairy goats. The result showed that the goats fed with purple Napier grass silage had the highest nutrient digestion; this is consistent with the study by Tian et al. [23], in which it was reported that the digestibility of Napier grass silage rich in anthocyanins had the highest values and was significantly different when compared to the other groups. This leads to a proper fermentation process within the rumen. Protein digestibility is also affected by the amount of protein present in the diet. The protein levels in the experimental food formulations were comparable. Digestibility in animals is based on the subject’s ability to digest microorganisms found in the rumen. The ability of microbes to digest feed that has passed through the stomach correlates positively with the number of microorganisms in the feed. In the study by AOAC [12], it was found that the digestibility in dairy goats fed with sticky corn stover silage, commercial purple corn pigment and anthocyanin-rich purple corn (AR) was higher than that of dairy goats fed with rice straw. The digestibility of nutrients can help to determine their usability. According to the study by Islam [7], feeding with grass silage can improve the performance of beef cattle.

### 4.3. Nitrogen Utilization

The results show that the goats fed with purple Napier grass silage had the highest nitrogen utilization. The study by Sanchez et al. [24] found that goats raised on Napier grass-based silage that was rich in anthocyanins and supplemented with 4% molasses and FeSO_4_ had high nitrogen retention. In [3], the authors also reported that goats fed with fresh purple Napier grass had the highest nitrogen balance. The study by Wang et al. [25] found that protein intake leads to enhanced nitrogen balance. It is worth mentioning that lower ruminal ammonia-N concentrations were found in goats fed a purple Napier grass silage diet. This could be because the purple Napier grass silage provided more fermentable carbohydrates, which play an important role in increasing rumen microbes by converting rumen degradable crude protein (CP) into microbial protein. This is because the amount of ammonia nitrogen produced exceeds the rumen’s requirements. Excess ammonia is converted to urea and excreted by the animal in its urine. According to the findings of the above study, the goats fed with bright purple Napier grass produced more ammonia nitrogen in their rumens. However, the possibility that greater anthocyanin consumption decreased dietary protein solubility in the rumens, which in turn decreased rumen ammonia-N concentrations, cannot be excluded. In addition, the study by [11,22] discovered that anthocyanins can stimulate the release of nitrogen and carbohydrates from purple corn, which helps to improve the efficiency of microorganisms.

### 4.4. Rumen Fermentation

According to the study by Wanapat et al. [26], decreased rumen fermentation prevents microorganism enzymes from acting and from exercising all of their functions. The experimental group fed with Napier Pakchong-1 grass silage had higher ammonia at 2 and 4 h after feeding. According to [27], the optimal level of ammonia nitrogen for bacterial growth was 9.7–21.4 mg/dL. Furthermore, the study by Lee et al. [28] found that the optimal level was 17.6 milligrams per 100 mg. In addition, the level of ammonia nitrogen in the rumen rises, which correlates with an increase in nitrogen levels in the blood. The remaining ammonia is absorbed by the rumen wall and enters the bloodstream before entering the urea cycle via the liver. According to the study by Mensching et al. [29], the amount of BUN in a ruminant ranges from 5 to 25 mg/dL, depending on the animal. As a result, the amount of BUN indicates the efficiency level that is appropriate for the fermentation of protein content and non-protein nitrogenous compounds (NPNs) in the diet, as well as the protein loss from the rumen in the form of urea. Our experiment revealed that the urea nitrogen levels of the goats fed with all three fermented roughage formulas were within the standard range for goat urea nitrogen content. It was found that there was a lack of energy from fermentation when the crude protein in the food was excessive or highly degradable. When raising animals, ready-mixed feed helps the rumen to use feed more efficiently and improves digestion. When the pH values in the rumen are appropriate, the animal can achieve its full production potential, according to Kmicikewycz et al. [30]. Thus, according to Nur et al. [31], ready-mixed feed (TMR) is important for animals because it can help to keep the pH in the rumen more stable than when animals are provided with separate forms of feed.

### 4.5. Volatile Fatty Acid

The goats fed with purple Napier grass silage had improved total VFA concentration, likely because the anthocyanins played a regulatory role in the growth of bacterial populations in the rumen, as demonstrated by the increased relative abundance of the microbial population. This study found that the volatile fatty acid concentrations in the goats in our experiment were within the normal range, which was consistent with the findings of Gonzalez-Garcia et al. [32].

At 2 and 4 h after feeding, the goats fed with purple Napier grass silage had increased propionic acid because the bacteria in the rumen fermented the soluble carbohydrates, converting them into simple sugars. According to the study by Wang [33], these sugars are used as an energy source by microorganisms to grow and produce propionic acid. In the study by Oba [34], soluble carbohydrates in forage plants are quickly degraded in the rumen for the synthesis of volatile fatty acids. The goats fed with purple Napier grass had higher propionic acid levels. This is consistent with the finding of Gunun et al. [27], who found that feeding fodder high in sugar content increases microbial nitrogen flow and the rumen microorganisms’ efficiency, leading to improved nutrient digestibility.

Our findings reveal that the goats given Napier Pakchong-1 grass silage had greater levels of butyric acid than the other groups. According to Bionaz et al. [35], forages increased the acetic acid concentration in the rumen, which increased the amount of fat in the milk. In addition to being metabolized in the body and used as energy, acetic acid serves as a precursor to fat in milk and meat. Propionic acid is converted into glucose and used in the production of milk sugar. Butyric acid is converted into β-hydroxybutyrate. According to Nagaraja et al. [36], during absorption, the rumen wall produces fat in the mammary glands and adipose tissue. It was found that more acetic acid is produced when animals are fed roughage, silage, and mature plants with a high roughage content, due to the factors influencing volatile fatty acid production. High protein intake leads to increased production of butyric acid. According to Kumar et al. [37], the energy level in food influences the concentration of VFA, causing the concentration of TVFA acids to increase as the energy level increases. However, in our experiment, the protein was adjusted to fall within the same range so that it would not produce negative effects on the concentration of volatile fatty acids.

### 4.6. Microbial Population

The utilization of purple Napier grass silage on lactating dairy goats led to a greater microbial population as well as reduced protozoa and methanogen. This could be due to the high protein digestibility in purple Napier grass silage and because its ammonia-nitrogen values are within the appropriate range for the functioning of microorganisms in the rumen, allowing bacteria to work more efficiently. The microbial populations found in the goats that were fed with purple Napier grass silage belonged to the Protozoa and Methanogen groups. According to Zhang et al. [38], the number of protozoa in the rumen decreased as the starch levels in the feed increased. It also reduced the incidence of mastitis, which is consistent with the findings of Bradley [39], who reported that tannin supplementation could reduce the amount of *Streptococcus gyloticus* while promoting growth, utilizing nutrients, and fighting mastitis. According to the findings of Carberry et al. [40], tannin supplementation inhibits *Streptococcus gyloticus*, which improves nitrogen digestibility in sheep. The study by Varga [41] reported that *Streptococcus gyloticus* can reduce the incidence of mastitis in cattle. It can be argued that diet is the most significant factor influencing the microbial population in the rumen. The study by [42] included an environment in which the rumen microorganisms could not utilize fatty acids as an energy source. The presence of high levels of fat content in the diet reduces the growth performance of microorganisms, particularly in the digestion of fibrous feeds. As a result, according to Piantoni et al. [43], animals consuming a high fat diet can consume less feed. According to Kumar et al. [37], mangosteen peel supplement causes low rumen protozoa and can have a specific effect on rumen protozoa. The reduced relative abundance of methanogens in purple Napier grass silage goats could perhaps be explained by the higher anthocyanin components in the rumen fluid, which might inhibit methanogenesis by causing more electron exchanges.

### 4.7. Milk Quality

This study found that goats fed with PNS had the highest milk production and milk composition and a decreased somatic cell count. According to Onjai-Uea et al. [3], goats fed only PNS produced milk with more components. In addition, the number of somatic cells in their milk decreased. This could be attributed to the amount of dry matter and nutrients received. According to Lemosquet et al. [44], the type of feed and dry matter consumed influences the composition of the milk, and the quality of food influences the synthesis of milk components. In the study by Dong et al. [45], it is shown that milk yield and protein in milk are related to an increase in dry matter content (DMI). According to the study by Mader et al. [46], this is because propionic acid is a precursor to lactose synthesis in milk. In the study by Gonzalez-Garcia et al. [32], it was found that roughage fed to animals in the Napier grass silage group had no negative impact on milk production or milk composition in the ruminants; this is consistent with the finding of Lounglawanc et al. [2]. Because roughage contains a high percentage of cellulose, it can boost fat levels in milk and can increase the concentration of acetic acid in the rumen, affecting the fat synthesis process in milk. The study by Leatherwood [47] reported a decrease in somatic cell values, which suggests that anthocyanins have antibacterial properties (including the ability to destroy bacterial cell walls). Hosoda et al. [48] identified three types of roughage sources—dried alfalfa, dried oats, and corn silage—that do not have a negative effect on the chemical or physical composition of goat milk. However, they did affect the composition of milk fat because the amount of fiber in feed influences the amount of fat in milk.

### 4.8. Plasma Antioxidant

Blood biochemical indices can indicate an animal’s health status, including dietary absorption and metabolism. In the current study, the highest levels of total antioxidant, SOD, and GSH and reduced MDA were shown in comparison with the other treatments; purple Napier grass may contain more secondary compounds, such as anthocyanins, which are glycosides of anthocyanidins. Anthocyanins are antioxidants that naturally appear in the pigments of plants used as food sources for animals. It is believed that the higher-level anthocyanins (such as peonidin-3-Oglucoside, malvidin-3-O-glucoside, and malvidin) provided better protection against free radicals and decreased inflammation. According to Hosoda et al. [49], they function as Phyto acyl antioxidants or as an antibacterial agent with a stronger effect on in vitro fermentation, and they increase aspartate aminotransferase (AST) and superoxide dismutase activity and SOD in plasma [21]. In a study by Cui et al. [50], anthocyanins and 3-deoxyanthocyanidins increased plasma activity in SOD, an important antioxidant enzyme, in sheep. According to a study by Hosoda et al. [21], fermented mulberry leaves produce high-quality animal feed or bioactive pharmaceuticals that benefit ruminant health. According to study a by Tian et al. [11], feeding with anthocyanin-rich corn silage reduced MDA. Increasing antioxidant enzyme activity in blood may result in a decrease in MDA levels by optimizing the rumen and its rumen bilayers for the dietary bioactive chemicals produced from purple Napier grass silage through a greater fluidity of the membrane. Considering that SOD is the first line of defense against ROS scavenging in intracellular enzymes, we hypothesized, based on the current data, that the increased level of anthocyanin in the purple Napier grass silage diet functions as an electron donor, lowering the buildup of reactive oxygen species (ROS). In contrast, the CAT and GSH activation implies that the concentration of superoxide anion radicals during dismutation exceeded the threshold, resulting in reactive oxygen species (ROS) being enzymatically scavenged by catalase (CAT) or glutathione (GSH) before being reduced to water. It appears that superoxide dismutase (SOD) and GSH worked together to enhance ROS scavenging. Consequently, the lower malondialdehyde (MDA) values and higher total antioxidant capacity (TAC) levels were induced not only by the antioxidative activity of anthocyanins but also by the increased expression of SOD, TAC, and GSH.

## 5. Conclusions

Feeding with Purple Napier grass silage significantly enhanced the growth performance, nutrient digestibility, nitrogen utilization, rumen fermentation parameters, microbial population, milk quality, and plasma antioxidant activity in goats. This enhancement in animal productivity was partly due to higher rumen fermentation activity, as evidenced by the higher plasma antioxidant activity. Concurrently, the higher anthocyanin content of the purple Napier grass silage diet reduced stress by increasing plasma antioxidant activity; this was evidenced by the lower MDA concentrations and higher total antioxidant, SOD, and GSH concentrations in the blood plasma, which resulted in improved health. The goats fed the purple Napier grass silage diet had lower rumen ammonia nitrogen and a relative abundance of methanogens. Collectively, the findings of this study could provide insights into the nature of agricultural and roughage-based diets, as their health-improving features can offer an alternative to the use of synthetic growth promoters in the production of sustainable animal feed and animal products.

## Figures and Tables

**Table 1:** Chemical composition of grass species and effect of harvest time on the nutritional value of sweet grass, Napier Pakchong-1 grass and, purple Napier grass in silage form.

Items	Napier Pakchong-1	Sweet Grass	Purple Napier
Day	60	60	60
Chemical composition (% DM)			
DM	22.75	24.72	22.85
Ash	11.93	9.25	9.87
CP	10.59	11.82	10.58
EE	3.92	3.46	3.12
CF	39.24	29.40	31.18
NDF	72.16	59.08	64.96
ADF	40.45	40.57	45.39
ME, kJ/g DM	2460.04	2020.73	2518.10
DE, kJ/g DM	3000.05	3005.25	3070.85
GE, kJ/g DM	3800.25	4081.60	4100.60

Note: % DM: percentage of dry matter; % CP: percentage of crude protein; % EE: percentage of fat; % CF: percentage of crude fiber; % NDF: percentage of neutral detergent fiber; % ADF: percentage of acid detergent fiber; ME: metabolizable energy (ME = 0.82 × DE), DE: digestible energy; GE: gross energy.

**Table 2:** Ingredients and chemical compositions of Napier Pakchong-1, sweet grass and purple Napier grass silage in diets of dairy goats.

		Treatment	
Item	NPS	SGS	PNS
Ingredient (% DM)		On dry basis%	
Napier Pakchong-1 grass silage	84.40	-	-
Sweet grass silage	-	84.40	-
Purple Napier grass silage	-	-	84.40
Full-fat soybean	5.00	5.00	5.00
Corn	4.00	4.00	4.00
Rice bran	4.00	4.00	4.00
Oil palm	2.00	2.00	2.00
Calcium phosphate	0.40	0.40	0.40
Salt	0.20	0.20	0.20
Total	100	100	100
Chemical composition			
DM	28.57	26.28 On dry basis%	30.49
Ash	13.25	12.25	10.70
CP	16.15	16.49	16.21
EE	4.72	4.66	4.19
CF	15.78	16.09	22.06
NDF	50.85	49.39	48.80
ADF	27.99	24.29	34.27
TDN	457.29	498.11	522.60
Metabolizable energy, Mcal/kg DM	16.53	18.00	18.89

DM, dry matter; CP, crude protein; NDF, neutral detergent fiber; ADF, acid detergent fiber; CF, crude fiber; EE, ether extract; NPS = Napier Pakchong-1 grass silage; SGS = sweet grass silage; PNS = purple Napier grass silage; TDN = (%) DCP + DNFC) + DEE × 2.25 + (DNDF); estimated by the equation ME (Mcal/kg DM) = (TDN × 0.04409 × 0.82).

**Table 3:** Effect of utilization of purple Napier grass silage on growth performance, feed intake, and nutrient intake in dairy goats.

Item	Treatment			SEM	*p* Value
	NPS	SGS	PNS		
Initial weight (kg)	43.35	43.25	43.30	0.02	0.18
Final weight (kg)	45.50 ^c^	46.51 ^b^	47.72 ^a^	0.25	<0.0001
Weight change	2.15 ^c^	3.26 ^b^	4.42 ^a^	0.25	<0.0001
Milk production (g/day)	1171.60 ^c^	1524.18 ^b^	1650.33 ^a^	13.81	<0.0001
Voluntary feed intake					
g DM/d	1312.76 ^c^	1443.43 ^b^	1805.97 ^a^	29.36	<0.0001
% BW	2.89 ^c^	3.10 ^b^	3.78 ^a^	0.10	<0.0001
g/kg BW^0.75^	52.76 ^c^	54.70 ^b^	59.24 ^a^	0.90	0.002
Nutrient intake g DM/d					
OMI	1138.82 ^c^	1266.61 ^b^	1612.73 ^a^	16.26	<0.0001
CPI	212.27 ^c^	238.02 ^b^	292.75 ^a^	2.36	0.0007
EEI	61.96 ^c^	67.26 ^b^	75.67 ^a^	1.28	0.0007
NDFI	667.54 ^c^	712.91 ^b^	881.31 ^a^	8.47	<0.0001
ADFI	367.44 ^c^	350.61 ^b^	618.91 ^a^	7.53	0.0003

NPS, Napier Pakchong-1 grass silage; SGS, sweet grass silage; PNS, purple Napier grass silage; a, b, c indicate a significant difference (*p* < 0.05) within the same row; g DM/d, daily intake of dry matter; % BW, edible quantity per body weight per day; g/kg BW^0.75^.

**Table 4:** The effect of utilization of purple Napier grass silage on nutrient digestibility in dairy goats.

Item	Treatment			SEM	*p* Value
	NPS	SGS	PNS		
Apparent Digestibility, % of Intake					
DDM	63.24 ^c^	69.19 ^b^	74.68 ^a^	1.80	0.0046
DOM	65.31 ^c^	69.65 ^b^	76.43 ^a^	1.72	0.0019
DCP	67.49 ^c^	75.43 ^b^	79.85 ^a^	1.87	0.0004
DEE	85.44 ^c^	91.46 ^a^	93.32 ^b^	1.83	0.0008
DNDF	79.38 ^c^	83.88 ^b^	87.74 ^a^	1.32	0.0051
DADF	37.70 ^c^	48.28 ^b^	57.15 ^a^	2.97	0.0012

NPS, Napier Pakchong-1 grass silage; SGS, sweet grass silage; PNS, purple Napier grass silage; a, b, c indicate a significant difference (*p* < 0.05) within the same row.

**Table 5:** Effect of utilization of purple Napier grass silage on nitrogen utilization in dairy goats.

Item	Treatment			SEM	*p* Value
	NPS	SGS	PNS		
N intake (g/d)	33.96 ^b^	38.08 ^b^	46.84 ^a^	0.94	<0.0001
N faces (g/d)	9.52 ^c^	10.77 ^b^	11.66 ^a^	0.35	0.0070
N urine (g/d)	6.18	7.12	5.95	2.67	0.1715
N absorption (g/d)	24.44 ^c^	27.31 ^b^	35.18 ^a^	0.23	0.0014
N absorption (%)	71.97 ^b^	71.72 ^a^	75.11 ^a^	1.13	0.0158
N retention (g/d)	18.26	20.19	29.23	0.53	0.3045
N retention (%)	53.77 ^b^	53.02 ^b^	62.40 ^a^	0.92	0.0101

NPS, Napier Pakchong-1 grass silage; SGS, sweet grass silage; PNS, purple Napier grass silage; a, b, c indicate a significant difference (*p* < 0.05) within the same row.

**Table 6:** Effect of utilization of purple Napier grass silage on rumen fermentation in dairy goats.

Items	NPS	SGS	PNS	SEM	*p* Value
Levels of pH					
0 h	6.36	6.53	6.29	0.08	0.58
2 h	6.94	6.76	6.91	0.06	0.52
4 h	6.56	6.54	6.53	0.06	0.98
Ammonia nitrogen mg/dL					
0 h	15.25	15.20	15.38	0.05	0.26
2 h	20.21 ^a^	17.37 ^c^	19.18 ^b^	0.42	<0.0001
4 h	19.18 ^a^	16.17 ^c^	18.21 ^b^	0.44	<0.0001
BUN mg/dL					
0 h	15.61	15.45	15.18	0.09	0.14
2 h	20.59 ^a^	18.60 ^c^	19.71 ^b^	0.21	<0.0001
4 h	18.63 ^a^	16.65 ^c^	17.62 ^b^	0.21	<0.0001

a, b, c indicate a significant difference (*p* < 0.05) within the same row; BUN, blood urea nitrogen.

**Table 7:** Effect of utilization of purple Napier grass silage on volatile fatty acids in dairy goats.

Items	NPS	SGS	PNS	SEM	*p* Value
Acetic acid (molar proportion, %)					
0 h	63.77	63.71	64.64	0.22	0.15
2 h	64.15 ^c^	64.24 ^b^	64.86 ^a^	0.21	0.01
4 h	64.39	64.13	64.72	0.11	0.06
Mean	64.10 ^c^	64.46 ^b^	64.74 ^a^	0.10	0.02
Propionic acid (molar proportion, %)					
0 h	20.85	21.01	20.88	0.11	0.87
2 h	22.40 ^a^	22.08 ^c^	22.24 ^b^	0.34	<0.0001
4 h	20.93 ^b^	21.01 ^b^	21.39 ^a^	0.14	0.03
Mean	21.39 ^b^	21.37 ^b^	21.50 ^a^	0.16	<0.0001
Butyric acid (molar proportion, %)					
0 h	12.85	12.74	11.90	0.28	0.36
2 h	14.75 ^a^	14.50 ^b^	14.65 ^a^	0.21	<0.0001
4 h	15.70	16.03	15.72	0.08	0.20
Mean	14.43	14.42	14.09	0.11	0.35
Acetic acid: Propionic					
0 h	3.05	3.03	3.09	0.02	0.38
2 h	2.80 ^b^	2.97 ^a^	2.66 ^c^	0.05	<0.0001
4 h	3.08	3.05	2.98	0.02	0.17
Mean	2.97 ^b^	3.02 ^a^	2.91 ^c^	0.02	0.03
Total VFA (mmol/L)					
0 h	97.47	97.46	97.42	0.34	0.19
2 h	101.03 ^a^	100.82 ^c^	101.75 ^b^	1.29	<0.0001
4 h	101.02 ^a^	101.17 ^c^	101.83 ^b^	1.83	<0.0001
Mean	99.84 ^a^	99.82 ^c^	100.33 ^b^	1.06	<0.0001

a, b, c indicate a significant difference (*p* < 0.05) within the same row.

**Table 8:** Effect of utilization of purple Napier grass silage on microbial population in dairy goats.

Items	NPS	SGS	PNS	SEM	*p* Value
Total bacteria (lg10 copies/mL)					
0 h	2.42	2.31	2.78	0.09	0.13
2 h	4.32 ^b^	2.82 ^c^	5.58 ^a^	0.41	<0.0001
4 h	3.41 ^b^	2.49 ^c^	4.35 ^a^	0.27	<0.0001
Mean	3.39 ^b^	2.54 ^c^	4.24 ^a^	0.25	<0.0001
*Butyrivibrio fibrisolven* (lg10 copies/mL)					
0 h	1.25	1.22	1.26	0.01	0.39
2 h	3.31 ^b^	4.39 ^c^	6.50 ^a^	0.48	<0.0001
4 h	2.20 ^b^	3.15 ^c^	4.56 ^a^	0.34	<0.0001
Mean	2.25 ^b^	2.92 ^c^	4.10 ^a^	0.27	<0.0001
*Fibrobacter succinogenes* (lg10 copies/mL)					
0 h	1.75	1.76	1.71	0.01	0.09
2 h	2.56 ^c^	4.14 ^b^	6.43 ^a^	0.56	<0.0001
4 h	2.29 ^c^	3.41 ^b^	4.88 ^a^	0.38	<0.0001
Mean	2.20 ^c^	3.10 ^b^	4.34 ^a^	0.31	<0.0001
*Ruminococcus albus* (lg10 copies/mL)					
0 h	5.24	5.17	5.45	0.06	0.10
2 h	7.31 ^b^	6.40 ^c^	9.24 ^a^	0.42	<0.0001
4 h	6.33 ^b^	5.25 ^c^	8.65 ^a^	0.50	<0.0001
Mean	6.29 ^b^	5.61 ^c^	7.78 ^a^	0.32	<0.0001
*Ruminococcus flavefacises* (lg10 copies/mL)					
0 h	2.32	2.43	2.45	0.08	0.85
2 h	5.28 ^b^	4.53 ^c^	6.52 ^a^	0.30	0.0004
4 h	4.20 ^b^	3.56 ^c^	5.40 ^a^	0.28	0.0002
Mean	3.93 ^b^	3.50 ^c^	4.79 ^a^	0.19	<0.0001
*Streptococcus bovis* (lg10 copies/mL)					
0 h	3.35	3.32	3.47	0.04	0.27
2 h	7.61 ^b^	5.58 ^c^	9.56 ^a^	0.58	<0.0001
4 h	6.40 ^b^	4.61 ^c^	8.84 ^a^	0.62	<0.0001
Mean	5.79 ^b^	4.50 ^c^	7.29 ^a^	0.40	<0.0001
Protozoa (lg10 copies/mL)					
0 h	2.46	2.58	2.59	0.11	0.89
2 h	5.27 ^b^	6.22 ^a^	4.50 ^c^	0.26	0.0002
4 h	4.43 ^b^	5.52 ^a^	3.30 ^c^	0.33	<0.0001
Mean	4.05 ^b^	4.77 ^a^	3.46 ^c^	0.19	0.0001
Methanogen (lg10 copies/mL)					
0 h	3.57	3.46	3.51	0.01	0.92
2 h	5.51 ^b^	6.64 ^a^	4.27 ^c^	0.35	0.0001
4 h	5.24 ^b^	6.34 ^a^	3.53 ^c^	0.42	0.0003
Mean	4.77 ^b^	5.48 ^a^	3.77 ^c^	0.26	0.0003

a, b, c indicate a significant difference (*p* < 0.05) within the same row.

**Table 9:** Effect of utilization of purple Napier grass silage on milk quality in dairy goats.

Item	NPS	SGS	PNS	SEM	*p* Value
DMI (g/d)	1130.70 ^c^	1163.81 ^b^	1208.54 ^a^	7.893	<0.0001
Milk production					
Milk yield (kg/day)	1.05 ^b^	0.97 ^b^	1.22 ^a^	0.02	<0.0001
3.5% FCM (kg/day)	0.88 ^b^	0.91 ^b^	1.14 ^a^	0.03	<0.0001
Protein (g/day)	29.45 ^b^	31.42 ^b^	40.47 ^a^	1.33	<0.0001
Fat (g/day)	31.17 ^b^	32.34 ^b^	41.61 ^a^	1.33	<0.0001
Lactose (g/day)	39.47 ^b^	41.25 ^b^	56.59 ^a^	2.09	<0.0001
Milk solid not fat (g/day)	64.16 ^c^	68.28 ^b^	93.50 ^a^	5.38	<0.0001
Total solid (g/day)	98.95 ^c^	117.56 ^b^	144.89 ^a^	3.50	<0.0001
Milk composition					
pH	6.57	6.58	6.60	0.01	0.283
Protein (%)	3.35 ^c^	3.55 ^b^	3.73 ^a^	0.04	0.0001
Fat (%)	3.55 ^b^	3.65 ^b^	3.83 ^a^	0.005	0.003
Lactose (%)	4.49 ^c^	4.66 ^b^	5.21 ^a^	0.09	<0.0001
Milk solids not fat (%)	7.71 ^c^	8.49 ^b^	9.51 ^a^	0.15	<0.0001
Total solid (%)	11.26 ^c^	12.14 ^b^	13.34 ^a^	0.23	<0.0001
SCC (cells × 10^6^/mL)	1.74 ^a^	1.28 ^b^	0.98 ^c^	0.08	<0.0001

a, b, c indicate a significant difference (*p* < 0.05) within the same row; SCC, somatic cell count.

**Table 10:** Effect of utilization of purple Napier grass silage on plasma antioxidant in dairy goats.

Items	NPS	SGS	PNS	SEM	*p* Value
Total antioxidants (nmol/uL)					
0 h	1.43	1.36	1.48	0.04	0.62
2 h	3.05 ^b^	1.96 ^c^	3.66 ^a^	0.03	<0.0001
4 h	3.14 ^b^	2.64 ^c^	3.63 ^a^	0.04	<0.0001
Mean	2.54 ^b^	1.98 ^c^	2.92 ^a^	0.0089	<0.0001
SOD (inhibition rate%)					
0 h	80.44	80.36	80.57	0.08	0.47
2 h	88.60 ^b^	86.05 ^c^	94.75 ^a^	0.38	<0.0001
4 h	86.25 ^b^	81.58 ^c^	92.23 ^a^	1.63	<0.0001
Mean	85.10 ^b^	82.66 ^c^	89.18 ^a^	0.19	<0.0001
GSH (units/mL)					
0 h	60.49	60.42	60.48	0.14	0.07
2 h	70.79 ^b^	63.91 ^c^	74.04 ^a^	0.42	<0.0001
4 h	64.16 ^b^	62.55 ^c^	69.58 ^a^	0.90	<0.0001
Mean	65.15 ^b^	62.29 ^c^	68.04 ^a^	0.13	<0.0001
MDA (µg/mL)					
0 h	30.38	30.52	30.24	0.03	0.94
2 h	39.33 ^b^	42.50 ^a^	30.84 ^c^	0.79	<0.0001
4 h	32.43 ^b^	39.43 ^a^	30.75 ^c^	0.58	<0.0001
Mean	34.05 ^b^	37.48 ^a^	30.61 ^c^	0.12	<0.0001

a, b, c indicate a significant difference (*p* < 0.05) within the same row; SOD, superoxide dismutase; GSH, glutathione peroxidase; MDA, malondialdehyde.

## Data Availability

Data are contained within the article.
